# Design and Characterization of Prodrugged Anti‐CTLA‐4 Antibodies

**DOI:** 10.1002/cbic.202500304

**Published:** 2025-10-28

**Authors:** Sayumi Yamazoe, Mary Huber, Srikanth Kotapati, Rahima Akter, Aarti Jashnani, Suprit Deol, Christine Bee, John Engelhart, Yam B. Poudel, Stanley Krystek, John Haugner, Mohan Srinivasan, Arvind Rajpal, Yong Zhang, Pavel Strop, Chetana Rao

**Affiliations:** ^1^ Research and Development Bristol Myers Squibb Redwood City CA 94063 USA; ^2^ Research and Development Bristol Myers Squibb Cambridge MA 02141 USA

**Keywords:** antibody‐drug‐conjugate, immune checkpoint inhibitors, pharmacokinetics, prodrugging, site‐specific conjugation

## Abstract

Therapeutic antibodies are widely used to treat diseases like cancer and inflammatory conditions by binding with high specificity to their molecular targets. Masking is a strategy to mitigate undesirable activity in nontarget tissues, improving safety and pharmacokinetic (PK) profiles by reducing target‐mediated drug disposition. In this article, masking of an anti‐CTLA‐4 antibody by conjugating a large PEG molecule to specific sites on the antibody is explored. While anti‐CTLA‐4 immunotherapy benefits solid tumor treatment, its adverse events limit its utility. Multiple conjugation sites within the complementarity‐determining regions and adjacent framework regions are evaluated to attenuate CTLA‐4 binding. The optimal site for maximizing masking efficiency is identified, allowing for efficient bioconjugation and functional restoration upon exposure to a cleaving enzyme in the tumor microenvironment. The prodrugged antibody exhibits reduced binding to CTLA‐4 and Fc gamma receptors, high stability, and an extended half‐life in a mouse model. This technology has the potential to improve the PK profile and safety attributes of therapeutic antibodies.

## Graphical abstract

Masking an anti‐CTLA‐4 antibody by conjugating a large PEG molecule to specific sites is explored as a way of mitigating undesirable activity in nontarget tissues. This prodrugged antibody exhibits reduced binding to CTLA‐4 and Fc gamma receptors, demonstrated high stability, and had an extended half‐life in a mouse model, potentially improving the pharmacokinetic profile and safety attributes of therapeutic antibodies.

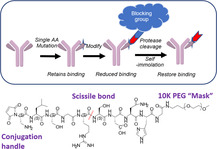

## 1 Introduction

Therapeutic antibodies have revolutionized the treatment of various diseases, particularly in oncology and immunology. Their ability to specifically target disease‐related antigens while minimizing off‐target effects makes them powerful agents in modern medicine.^[^ ^1^ ^]^ However, a primary challenge with conventional antibody therapy is that these antibodies can bind to their target antigens in both diseased and healthy tissues, leading to off‐target effects and toxicity.^[^ ^2^ ^]^ Prodrugging antibodies is a novel approach designed to enhance the precision and safety of antibody‐based treatments by reducing off‐site activity.^[^ ^3^ ^]^ Prodrugged antibodies minimize the likelihood of binding in nontargeted tissues, thereby preventing target‐mediated drug disposition (TMDD) and improving the pharmacokinetic (PK) profile of the treatment. These molecules allow for more precise targeting of diseased tissues, such as tumors, while sparing healthy tissues. This precision is particularly important in cancer therapy, where the goal is to maximize the therapeutic effect on cancer cells while minimizing damage to normal cells.^[^ ^4^ ^]^ By reducing off‐tumor on‐target toxicity, prodrugged antibodies can potentially allow for higher dosing of the therapeutic antibody, thereby increasing its efficacy without compromising safety. This broader therapeutic window can be crucial for achieving better clinical outcomes.

A prodrug is an inactive version of a therapeutic agent that can be converted at or near the target tissue or organ into the active therapeutic agent. Prodrugging is commonly achieved by covalently attaching a moiety that blocks the agent's activity. Removal of the blocking moiety at the target site by factors, such as low pH, specific enzymes, or anoxic conditions, restores the activity of the therapeutic agent.^[^ ^5^ ^]^ This masking can be achieved by attaching a peptide ‘mask’ to the antibody's antigen‐binding site, which blocks its ability to bind to the target.^[^ ^4^ ^b]^ The mask can be designed to be cleaved off by specific proteases that are abundant in the tumor microenvironment (TME) or diseased tissues. Once the mask is removed, the antibody is ‘activated’ and can bind to its target antigen, exerting its therapeutic effect.

We decided to use an anti‐CTLA‐4 antibody to evaluate our masking technology. The anti‐CTLA‐4 antibody has demonstrated significant therapeutic benefits in cancer treatment by enhancing immune responses against tumors.^[^ ^6^ ^]^ However, its use is associated with a range of immune‐related adverse events due to off‐tumor activity, which require careful monitoring and management to mitigate potential risks.^[^ ^7^ ^]^ Reducing off‐tumor activity could significantly help reduce toxicity and allow for higher dosing to achieve more effective treatment. To achieve this, we modified the antibody by site‐specific substitution of an amino acid in either the heavy or light chain variable region with a cysteine (Cys).^[^ ^8^ ^]^ The sulfhydryl group in the side chain of the substituted cysteine serves as a chemical handle for attaching a prodrugging moiety designed to interfere with the antibody's ability to bind to its antigen. The prodrugging moiety is attached via a cleavable linker that is processed by an enzyme overexpressed in the TME. We used the LSGX motif, which is recognized by matriptase, a protease upregulated in multiple tumors, to trigger cleavage in the TME.^[^ ^9^ ^]^ We leveraged a large PEG molecule to sterically hinder the binding potential while introducing a hydrophilic functionality known to improve the attributes of therapeutic antibodies by extending their PK and developability.^[^ ^10^ ^]^ Therefore, our masking molecule consists of maleimide for attachment to the antibody, a matriptase‐cleavable motif, and PEG. Our selection led to the discovery of prodrugged ipilimumab, which showed reduced binding and function in vitro. In vivo PK characterization of the prodrugged molecule in an MC38‐bearing mouse model suggested that the masked reagent is stable in circulation and undergoes cleavage in the TME. However, the study also showed off‐tumor cleavage once taken up by the tissues.

## 2 Results and Discussion

### 2.1 Prodrug Design and Bioconjugation

**Figure 1:**
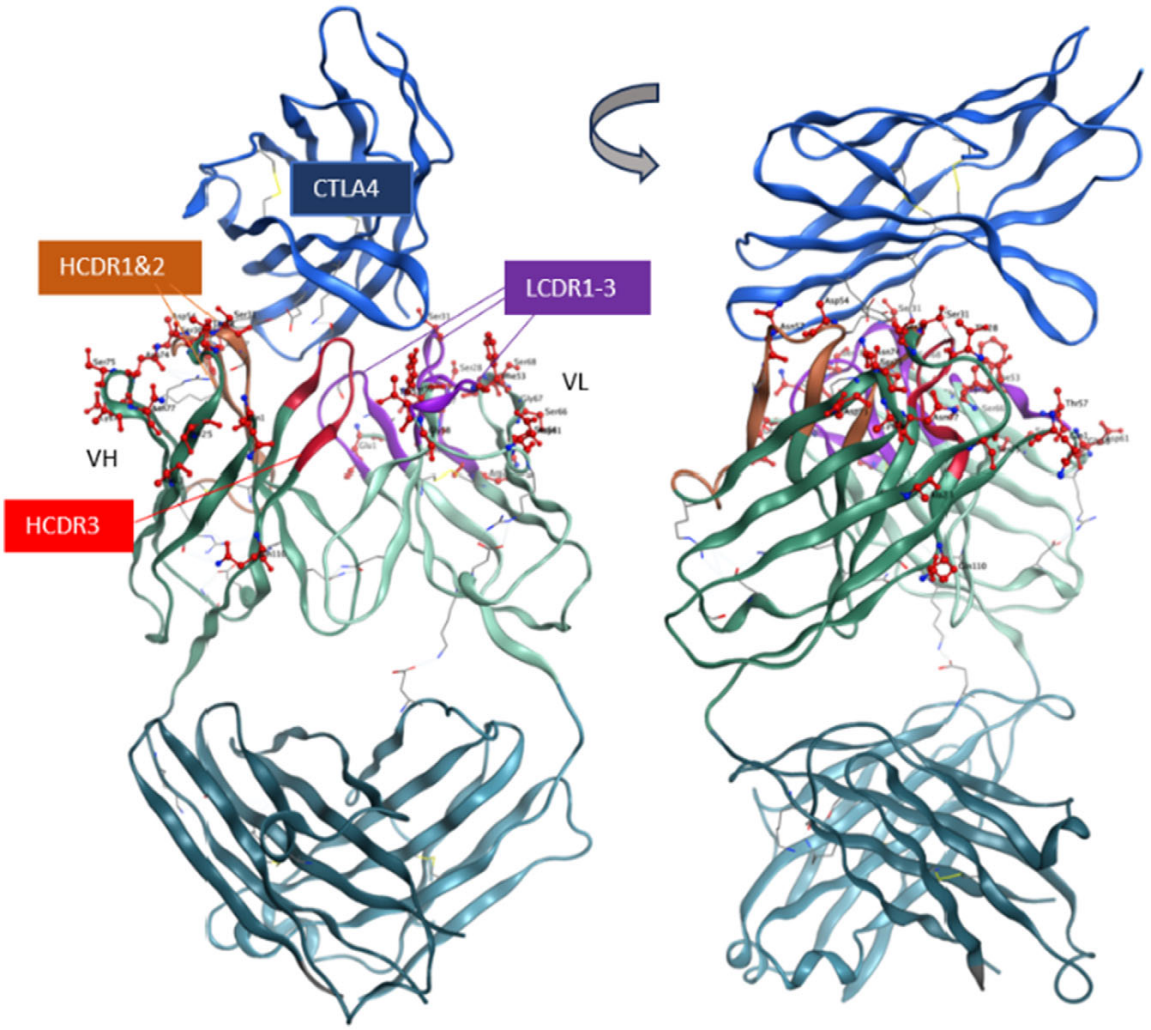
Bioconjugation sites evaluated for prodrugging the anti‐CTLA4 antibody. Residues highlighted in red were mutated to cysteine to enable site‐specific incorporation of the masking unit.

**Figure 2:**
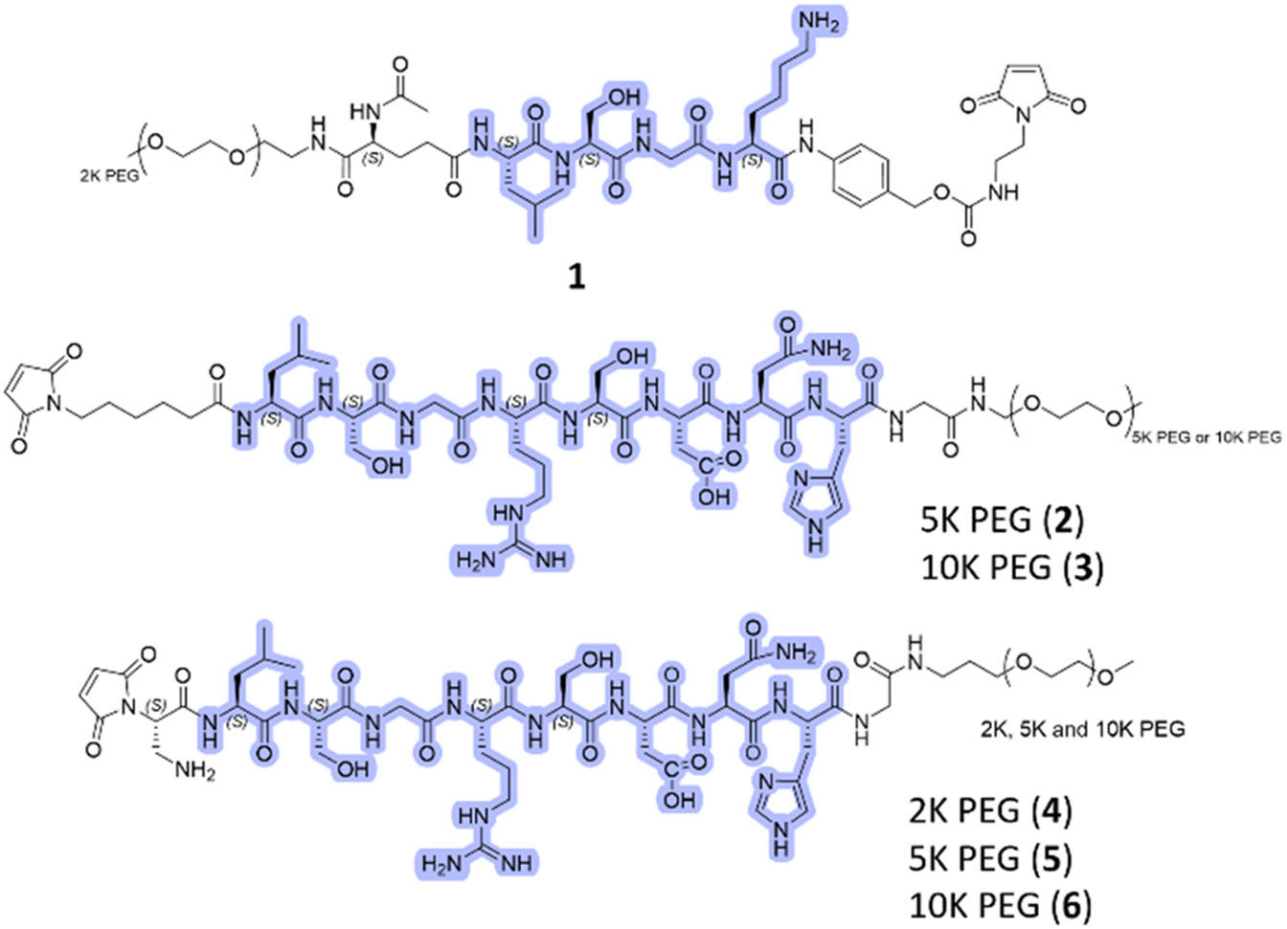
Structures of the masking reagent consist of: 1) a maleimide for conjugation, 2) a matriptase‐cleavable peptide (highlighted in purple), and 3) PEG to create steric hindrance, preventing the antibody from binding to the target.

We introduced unpaired cysteine residues into the complementarity‐determining regions (CDRs) or adjacent to the CDRs in both the light and heavy chains of ipilimumab to create genetic variants compatible with site‐specific bioconjugation (**Figure** **1**). Our goal was to identify the site that provides substantial attenuation in antigen binding and activity upon the introduction of a masking molecule, while allowing for restored activity upon demasking via linker cleavage. The masking reagent was designed to contain PEG2K, a cleavable LSGK motif, and a maleimide group for bioconjugation to the antibody (**Figure** **2**, 1). The engineered antibodies with unpaired cysteine residues contained covalent cysteine caps on the engineered cysteine residues via disulfides upon expression. Therefore, the conjugation process took place in three steps: 1) removal of the cap via partial reduction, 2) rebridging of interchain disulfides by oxidation, and 3) reaction with the masking reagent to introduce PEG at the selected site. Each step of the reaction was monitored by reversed‐phase (RP) high‐performance liquid chromatography (HPLC).^[^ ^11^ ^]^ Partial reduction with TCEP dissociated the heavy and light chains under denaturing conditions, and covalent bond formation with dhAA was evident by a retention time shift back to the prereduction state in RP‐HPLC. The introduction of the PEG reagent resulted in a major peak retention time shift in RP‐HPLC. The final conjugation step was conducted until the disappearance of the peak corresponding to the parental antibody, at which point, the conjugated product was purified by ion exchange chromatography to remove residual PEG reagent and high molecular weight species. If a high degree of aggregation was indicated at the reoxidation stage in RP‐HPLC, we decided not to progress to the conjugation step (T28, R24, Q27).

The conjugated products showed a band size shift in sodium dodecyl sulfate‐polyacrylamide gel electrophoresis, suggesting successful incorporation of the bulky unit (Figure S1, Supporting Information). Digestion of the prodrugged antibodies with human matriptase at 37 °C converted the masked molecules to their unmasked forms, which was confirmed by RP‐HPLC profile. Matriptase recognizes the LSGK motif and cleaves after the lysine residue, triggering a 1,6‐benzyl elimination at the para‐aminobenzyl carbamate group and subsequent decarboxylation to release a short 'stub’ attached to the antibody. The attachment of a small unit can be used to confirm the chemical modification of the antibody and drug‐to‐antibody ratio (DAR) by liquid chromatography mass spectrometry (LC‐MS), while the intact PEGylated molecules were not amenable to MS analysis due to poor ionization of the PEG unit. Additionally, we used size‐exclusion chromatography (SEC) to evaluate the monomer purity and integrity of the PEGylated molecules. We selected the prodrugged antibodies with ≥95% monomer purity and a DAR of 1.8 or above for further investigation (**Table** **1**). To evaluate the characteristics of both masked and unmasked formats and assess the shift in binding and function provided by masking, we also produced and purified the cleaved products, which were characterized by MS and SEC to ensure minimal heterogeneity (**Table** **2**).

**Table 1:** Conjugation site and conjugated products.

Site (Kabat)	Chain	Position	Linker	Aggregation	DAR	Product
Q1	Heavy	N‐term	PEG2K (**1**)	>5%	ND	NA
A23	Heavy	FR	PEG2K (**1**)	<1%	1.96	A23‐2K **1**
S25	Heavy	FR	PEG2K (**1**)	<1%	1.5	NA
T28	Heavy	FR	NAa)	>5%	ND	NA
S30	Heavy	CDR H1	PEG2K (**1**)	>5%	ND	NA
S31	Heavy	CDR H1	PEG2K (**1**)	<1%	0.8	NA
D54	Heavy	CDR H2	PEG2K (**1**)	>5%	ND	NA
N57	Heavy	CDR H2	PEG2K (**1**)	4%	2	N57‐2K **1**
D73	Heavy	FR	PEG2K (**1**)	>5%	ND	NA
N74	Heavy	FR	PEG2K (**1**)	<1%	1.9	N74‐2K **1**
S75	Heavy	FR	PEG2K (**1**)	<1%	1.9	S75‐2K **1**
K76	Heavy	FR	PEG2K (**1**)	>5%	ND	NA
E1	Light	N‐term	PEG2K (**1**)	>5%	ND	NA
R24	Light	CDR L1	NAa)	>5%	ND	NA
S26	Light	CDR L1	PEG2K (**1**)	2.30%	2	S24‐2K **1**
Q27	Light	CDR L1	NAa)	>5%	ND	NA
S28	Light	CDR L1	PEG2K (**1**)	<1%	2	S28‐2K **1**
S31	Light	CDR L1	PEG2K (**1**)	1.50%	2	S31‐2K **1**
F53	Light	CDR L2	PEG2K (**1**)	4%	2	F53‐2K **1**
T57	Light	CDR L2	PEG2K (**1**)	3.30%	2	T57‐2K **1**
D61	Light	FR	PEG2K (**1**)	1.90%	1.95	D61‐2K **1**
S68	Light	FR	PEG2K (**1**)	<1%	1.96	S68‐2K **1**

a) Aggregation observed after reoxidation.

**Table 2:** Cleaved products after digestion with matriptase and characterization data.

Site (Kabat)	Chain	Position	Linker	Aggregation	DAR	Product
A23	Heavy	FR	PEG2K (**1**)	<1%	1.93	A23‐2K **1** cleaved
N57	Heavy	CDR H2	PEG2K (**1**)	4.30%	2	N57‐2K **1** cleaved
N74	Heavy	FR	PEG2K (**1**)	2.90%	1.9	N74‐2K **1** cleaved
S26	Light	CDR L1	PEG2K (**1**)	<1%	2	S26‐2K **1** cleaved
S28	Light	CDR L1	PEG2K (**1**)	3%	2	S28‐2K **1** cleaved
S31	Light	CDR L1	PEG2K (**1**)	1.70%	2	S31‐2K **1** cleaved
F53	Light	CDR L1	PEG2K (**1**)	3.40%	2	F53‐2K **1** cleaved
T57	Light	CDR L1	PEG2K (**1**)	4.00%	2	T57‐2K **1** cleaved
D61	Light	FR	PEG2K (**1**)	3.00%	1.95	D61‐2K **1** cleaved
S68	Light	FR	PEG2K (**1**)	<1%	1.96	S68‐2K **1** cleaved

### 2.2 Functional Characterization of Prodrugged Antibodies

**Figure 3:**
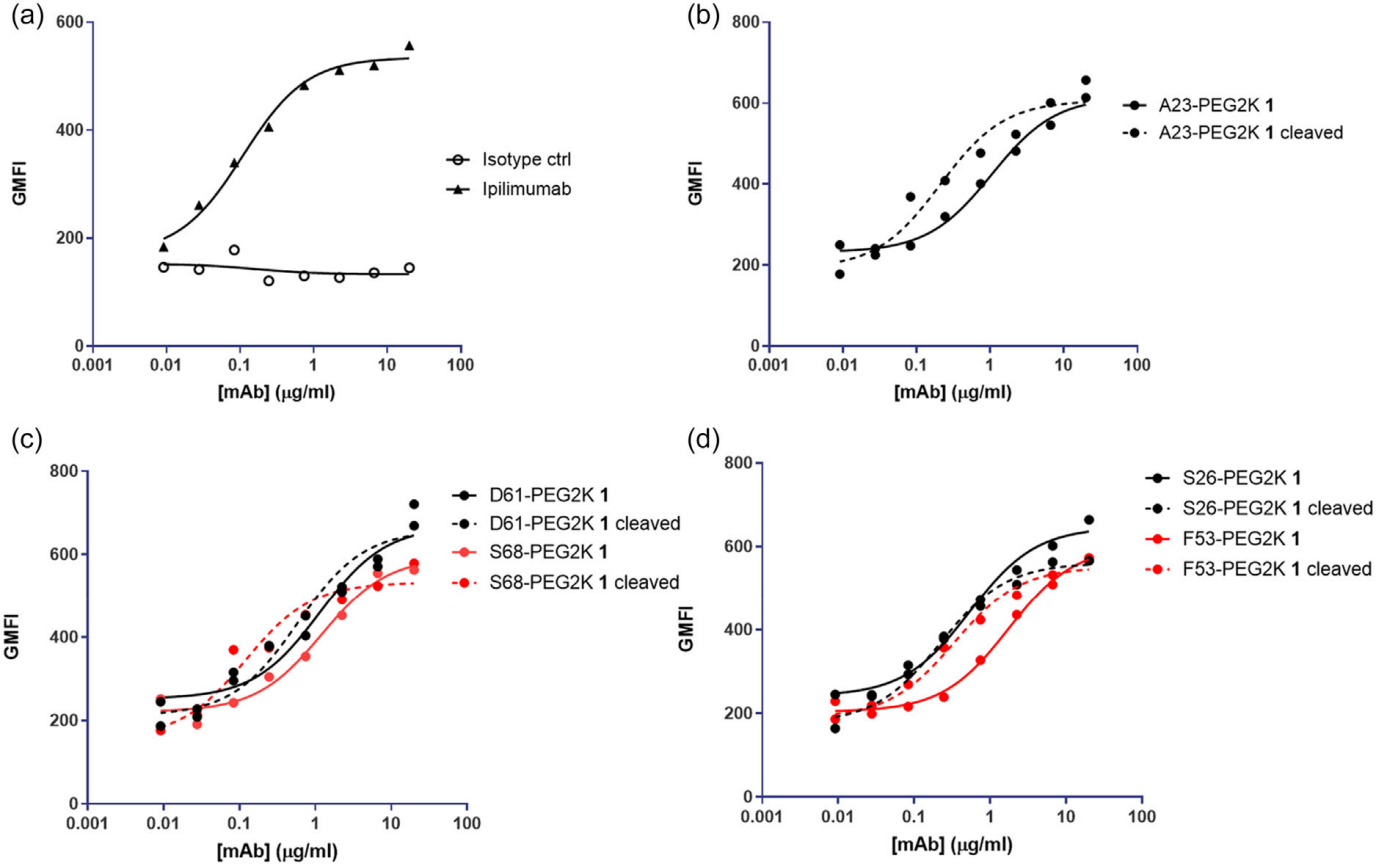
Binding to activated CD4+ T‐cells by prodrugged and unmasked formats of anti‐CLTA antibodies. Binding profiles were shown a) for Ipilimumab control, and for the constructs bearing cysteine mutation at b) heavy chain framework, c) light chain CDRs, and d) light chain framework.

Both prodrugged and unmasked antibodies were subjected to binding profile analysis on a cell line engineered to overexpress CTLA‐4. To our surprise, both masked and cleaved forms of the antibodies displayed equivalent levels of binding to the cell line, similar to ipilimumab (Figure S2, Supporting Information). A possible explanation for the lack of differentiation in binding on the engineered cell line could be the high level of CTLA‐4 expression on the surface, which presented challenges in testing attenuation. We then evaluated the binding of several constructs on primary CD4 T cells stimulated to express endogenous CTLA‐4. We observed a fold shift in fluoresence‐activated cell sorting (FACS) binding EC50 between intact and PEG‐cleaved antibodies, suggesting that the incorporation of the PEG reagent attenuated binding to the target. In the assay, the prodrugged antibodies bearing the masking reagent at positions A23, F53, and S68 demonstrated a prominent shift in binding EC50 upon PEG cleavage (**Figure** **3**, **Table** **3**).

**Table 3:** Binding of prodrugged and deprodrugged antibodies to CTLA4‐expressing cells. FACS EC50 values (ug mL^−1^) are shown.

Site of modification	PEG length	Binding on CTLA4‐transfectant			Binding on activated T cells		
		PEGylated antibody	Cleaved product	Ratio	PEGylated antibody	Cleaved product	Ratio
Parental mAb	NA	NA	0.275a)	NA	NA	0.118a)	NA
A23	2K	0.187	0.182	1.02	1.048	0.2102	4.99
N57	2K	1.899	0.782	2.42	ND	ND	
N74	2K	1.006	0.892	1.12	ND	ND	
S26	2K	0.134	0.315	0.42	0.5381	0.2207	2.438
S28	2K	0.538	0.665	0.82	ND	ND	
S31	2K	0.432	0.275	1.57	ND	ND	
F53	2K	0.171	0.115	1.48	1.743	0.3383	5.1522
T57	2K	0.532	0.569	0.93	ND	ND	
D61	2K	0.08	0.246	0.33	1.146	0.5873	1.952
S68	2K	0.25	0.272	0.92	1.219	0.1203	10.13

a) The FACS EC50 of the unmodified parental anti‐CTLA4 antibody was indicated as a benchmark.

Encouraged by the observed binding reduction, we decided to evaluate functional attenuation. The activity of prodrugged and deprodrugged antibodies was characterized by an in vitro functional assay using Staphylococcal enterotoxin B (SEB).^[^ ^12^ ^]^ SEB is a superantigen that strongly activates T cells and stimulates cytokine secretion. Fresh peripheral blood mononuclear cells (PBMCs) were isolated from two healthy human donors and treated with several concentrations of prodrugged and deprodrugged antibodies. Simultaneously, a suboptimal concentration of SEB was added to stimulate the cells. T‐cell activation was monitored by measuring the secretion of the cytokine interleukin (IL)‐2 after three days of incubation/treatment. Consistent with previous reports, ipilimumab exhibited dose‐dependent activity in different donors when compared to the IL‐2 secretion elicited by an isotype control antibody. When compared to the parental ipilimumab, the prodrugged antibodies showed reduced functional activity. Upon deprodrugging, there was approximately a threefold increase in IL‐2 secretion at the highest concentration of the deprodrugged antibodies, similar to the positive control of the unmodified CTLA‐4 antibody. This result confirms that reducing the binding of an antibody by attaching a blocking moiety reduces activity in a functional T cell assay and that such activity is restored upon removal of the blocking moiety. A varying degree of fold change in activity between the prodrugged and unmasked forms of antibodies was observed, depending on the site of PEG incorporation. Among all reagents evaluated, the variants with the masking reagent incorporated at A23, S26, S31, F53, and S68 demonstrated the most prominent dynamic range in activity between masked and unmasked forms (**Table** **4**). Among these, we decided to focus on the A23 and S68 variants, which have cysteine mutations in the framework region, providing broad applicability to human IgG1 in general.

**Table 4:** IL‐2 secretion assay. Fold shift of IL‐2 production AUC of each molecule compared to isotype control baseline was shown.

Site of modification	PEG length	IL‐2 secretion assay		
		PEGylated antibody	Cleaved product	Fold change in activity between the prodrugged and unmasked forms
A23	2K	1.23	1.74	1.41
N57	2K	1.13	1.35	1.19
N74	2K	1.31	1.35	1.03
S26	2K	1.41	1.83	1.29
S28	2K	1.54	1.22	0.79
S31	2K	0.97	1.59	1.63
F53	2K	1.03	1.82	1.76
T57	2K	1.28	1.41	1.10
D61	2K	1.03	1.24	1.20
S68	2K	1.16	1.82	1.56
Ipilimumab	NA	2.12	NA	NA

**Figure 4:**
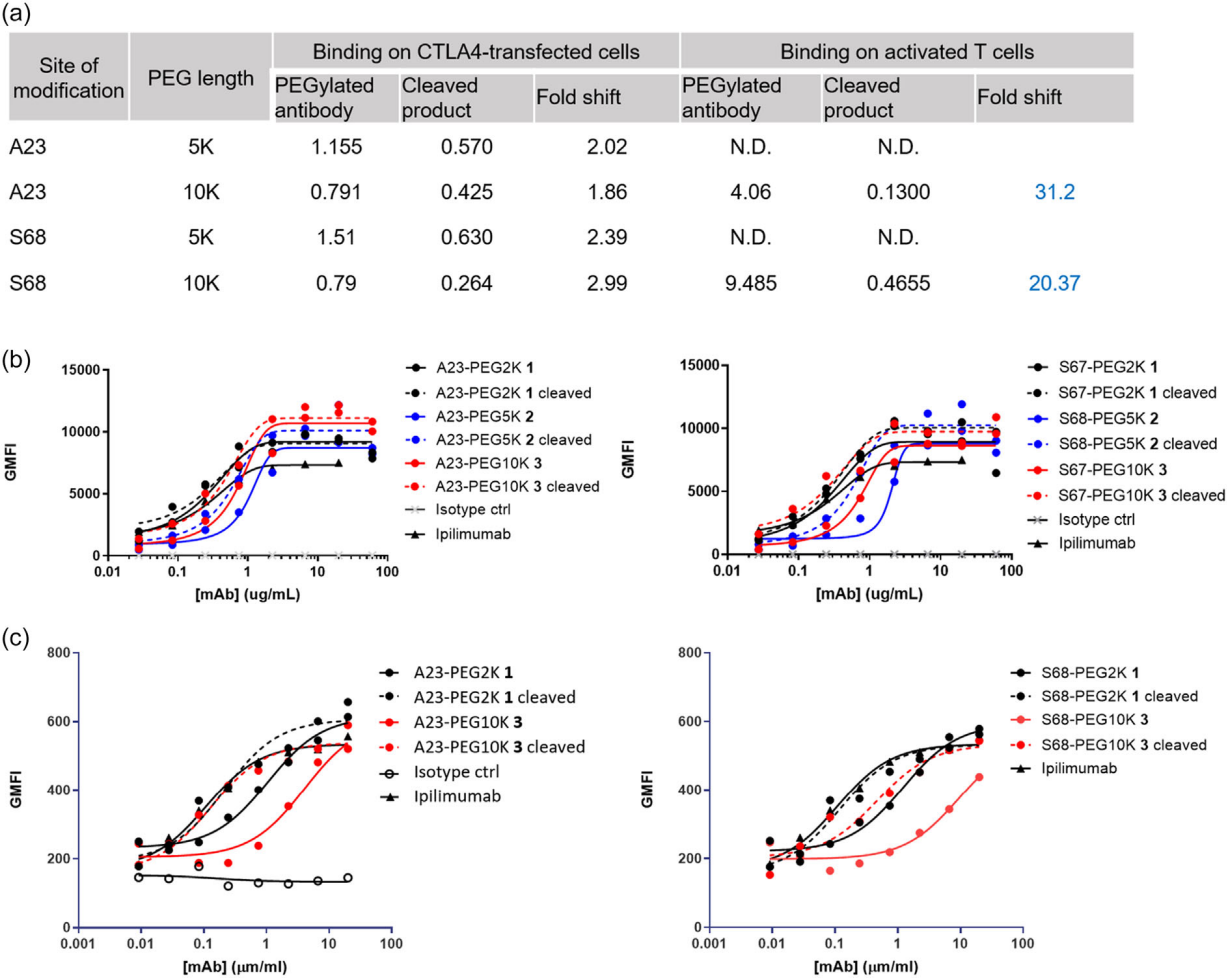
Binding profiles of prodrugged molecules bearing PEG5K and 10K reagents as well as their cleaved products on cell lines engineered to express CTLA4 and on activated T cells. a) EC50 values (ug mL^−^^1^) for bindings and the fold‐shift between the EC50 of the PEGylated antibody and the cleaved product. b,c) FACS binding profile shown (b) for CTLA4‐transfected cell line and (c) for activated T cells.

We decided to evaluate masking reagents with different PEG lengths using the A23 and S68 variants. The additional masking reagents were designed to contain a maleimide electrophile for bioconjugation to the antibody, a matriptase recognition peptide motif (LSGRSDNH), and PEG moieties of average sizes 5K and 10K (Figure 2, **2**, **3**).^[^ ^9^ ^b]^ Matriptase recognition results in protease cleavage after the Arg residue, releasing the antibody with a short ‘stub’ peptide. To validate the cleavage efficiency of the peptide motifs by matriptase, we measured cleavage kinetics using 7‐amino‐4‐methylcoumarin (AMC)‐modified peptides (LSGR and LSGK). The AMC‐functionalized peptides were incubated with human or mouse matriptase at neutral pH as well as at slightly acidic pH, which reflects the conditions commonly found in the TME.^[^ ^13^ ^]^ Both LSGR and LSGK peptides showed comparable cleavage kinetics with human and mouse matriptase (Figure S3, Supporting Information). While cleavage efficiency was generally reduced under acidic conditions, LSGR demonstrated relatively higher cleavage efficiency. The peptide was further subjected to a serum stability assay, which confirmed its stability in serum for up to 24 h (Figure S4, Supporting Information).The PEGylated molecules were synthesized analogously as described above for 2K PEG incorporation to the matriptase cleavable peptide unit. The cleaved counter molecules were produced by digestion of prodrugged molecules with human matriptase (Table S1, Supporting Information). The prodrugged antibodies generated with bulkier PEG reagents were subsequently evaluated for cellular binding on CTLA‐4‐expressing cells by flow cytometry. The prodrugged antibodies with 5K and 10K PEG demonstrated a reduced binding EC50 shift on CTLA‐4 transfectants, which was restored for unmasked antibodies (**Figure** **4**). We observed significant binding attenuation of the prodrugged antibodies on activated T cells for the 10K PEG‐incorporated variants (Figure 4). The level of binding attenuation was greater than what was observed with prodrugged species with 2K PEG. We then evaluated the ability of the prodrugged and deprodrugged antibodies to activate T cells using the SEB assay. The PEGylated antibodies failed to induce IL‐2 production by T cells in PBMCs, while the cleaved species exhibited activity, confirming the functional attenuation of the newly synthesized prodrugged antibodies. Although we observed improved cellular attenuation of these larger PEG‐modified antibodies, these reagents did not show enhanced functional attenuation in the T cell activation assay (**Table** **5**). The SEB assay was performed using PBMCs isolated from two donors to confirm the observations (Figure S5, Supporting Information).

**Table 5:** The activity of prodrugged and deprodrugged antibodies was characterized by an in vitro functional assay using SEB. Comparison of area under the dose–response curve (AUC) values of each molecule is shown. Isotype control was used as a baseline to calculate the AUC. The assay was performed with PBMCs isolated from two donors. Due to donor‐to‐donor variability, the AUC was calculated from the data of a single donor.

Site of modification	PEG length	SEB		
		PEGylated antibody	Cleaved product	Fold shift
A23	2K	1.23	1.74	1.41
A23	5K	1.13	1.76	1.55
A23	10K	1.06	1.71	1.58
S68	2K	1.16	1.82	1.56
S68	5K	0.86	1.62	1.88
S68	10K	1.04	1.51	1.45
Ipilimumab	NA	2.12	NA	NA

### 2.3 Serum Stability and Linker Design Modification

It is critical to prevent premature deconjugation in circulation before the antibodies are taken up by tumors to avoid off‐tumor binding. The serum stability of the prodrugged antibodies was assessed using immunocapture‐LC‐MS (IC‐LC‐MS). The test articles were incubated in serum at 37 °C for up to 4 days, and serum aliquots collected at various time points were analyzed by affinity capture LC‐MS. Briefly, the collected serum mixture was incubated with magnetic beads attached to the anti‐human Fc capture reagent. After immobilization on the beads, unbound components were washed out to enrich the protein of interest (Figure S6, Supporting Information). Finally, the antibodies captured on the beads were eluted and analyzed by LC‐MS after reduction to dissociate heavy and light chains.

**Figure 5:**
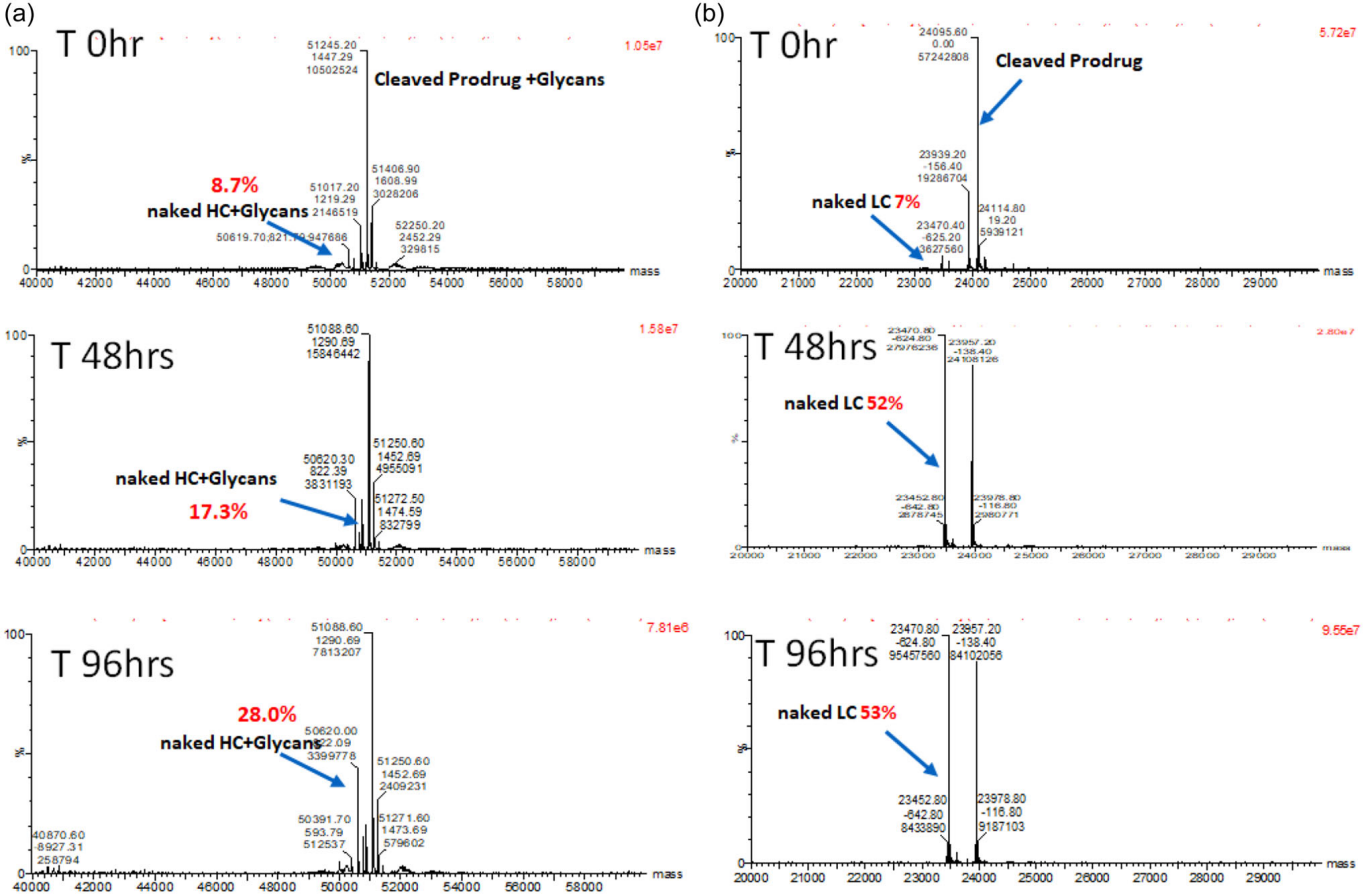
The serum stability was assessed using IC‐LC‐MS. Cleaved products were utilized to evaluate ex vivo serum stability and biotransformation. The unmasked molecule was added to SCID serum at a concentration of 50 µg mL^−1^ and incubated at 37 °C for up to 4 days. Serum aliquots were collected at various time points postspiking and analyzed by affinity capture LC‐MS. Deconvoluted mass spectra of samples from 0, 2, and 4 days were generated. The data for deprodrugged a) A23‐PEG5K and b) S78‐PEG5K molecules are presented.

**Figure 6:**
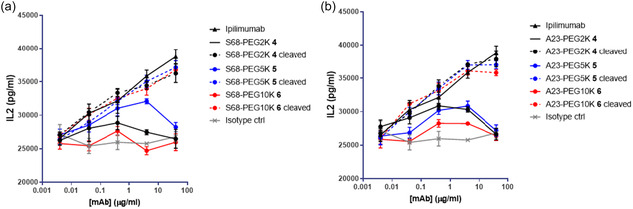
The activity of prodrugged and deprodrugged antibodies was characterized by an in vitro functional assay using SEB. a,b) IL‐2 production profile of prodrugged and unprodrugged molecules bearing masking group at (a) A23 and (b) S68 is shown.

Both prodrugged and unmasked antibodies for the A23 and S68 variants were subjected to the analysis. As expected, due to poor ionization, we were unable to detect conjugated species for the intact prodrugged product (Figure S7, S8, Supporting Information). On the contrary, analysis of deprodrugged antibodies provided meaningful signals after deconvolution (**Figure** **5**). For the S68 variant, we noted that the naked light chain signal increased over time, starting from 7% and reaching up to 53% at the 96‐hour time point (Figure 5). Thiosuccinimide bonds are known to undergo retro‐Michael reactions, particularly in the presence of thiol groups found in serum proteins.^[^ ^14^ ^]^ The observed deconjugation could be due to the thiol‐exchange reaction. Additionally, we observed hydrolysis events, likely due to thiosuccinimide ring opening reactions. The cleaved products also showed species without arginine, which could be due to the instability of peptide bonds upon cleavage and exposure of the C‐terminal arginine to serum proteinases. These biotransformation events, including deconjugation, hydrolysis, and loss of arginine, were observed for the A23 variant as well (Figure 5). The level of naked heavy chain increase was lower (28% at the 96‐hour time point) compared to the S68 variant. To fully prevent deconjugation, we introduced self‐hydrolyzable maleimide into the linker design (Figure 2) and evaluated conjugated species using the A23 and S68 antibody variants. Three different PEG lengths (2K, 5K, and 10K) were characterized (Figure 2, **4–** **6**). The conjugation and cleavage were produced and characterized as described above (Table S2, Supporting Information). Subsequently, the immunostimulatory activity of prodrugged and deprodrugged antibodies was evaluated by in vitro SEB assays. The prodrugged species demonstrated substantial functional attenuation for both the S68 and A23 variants bearing masking reagents with varying PEG lengths (**Figure** **6**). We decided to use the 10K PEG reagent for further characterization based on the lowest baseline activity observed.

### 2.4 Generation of Prodrugged Analog of Ipilimumab NF

The activity of ipilimumab in cancer therapeutics has confirmed the importance of CTLA‐4 blockade in immunotherapy. Based on the mechanism of action of ipilimumab, a nonfucosylated version, ipilimumab NF has been explored to enhance the antibody's activity.^[^ ^12^ ^,^ ^15^ ^]^ Ipilimumab‐NF is designed to have enhanced antibody‐dependent cellular cytotoxicity activity, which is expected to increase the potential for Treg depletion. Indeed, ipilimumab‐NF has been demonstrated to show increased IL‐2 secretion in peripheral mononuclear cells treated with the superantigen SEB in vitro and to display more potent antitumor activity in vivo compared to ipilimumab.^[^ ^16^ ^]^

**Figure 7:**
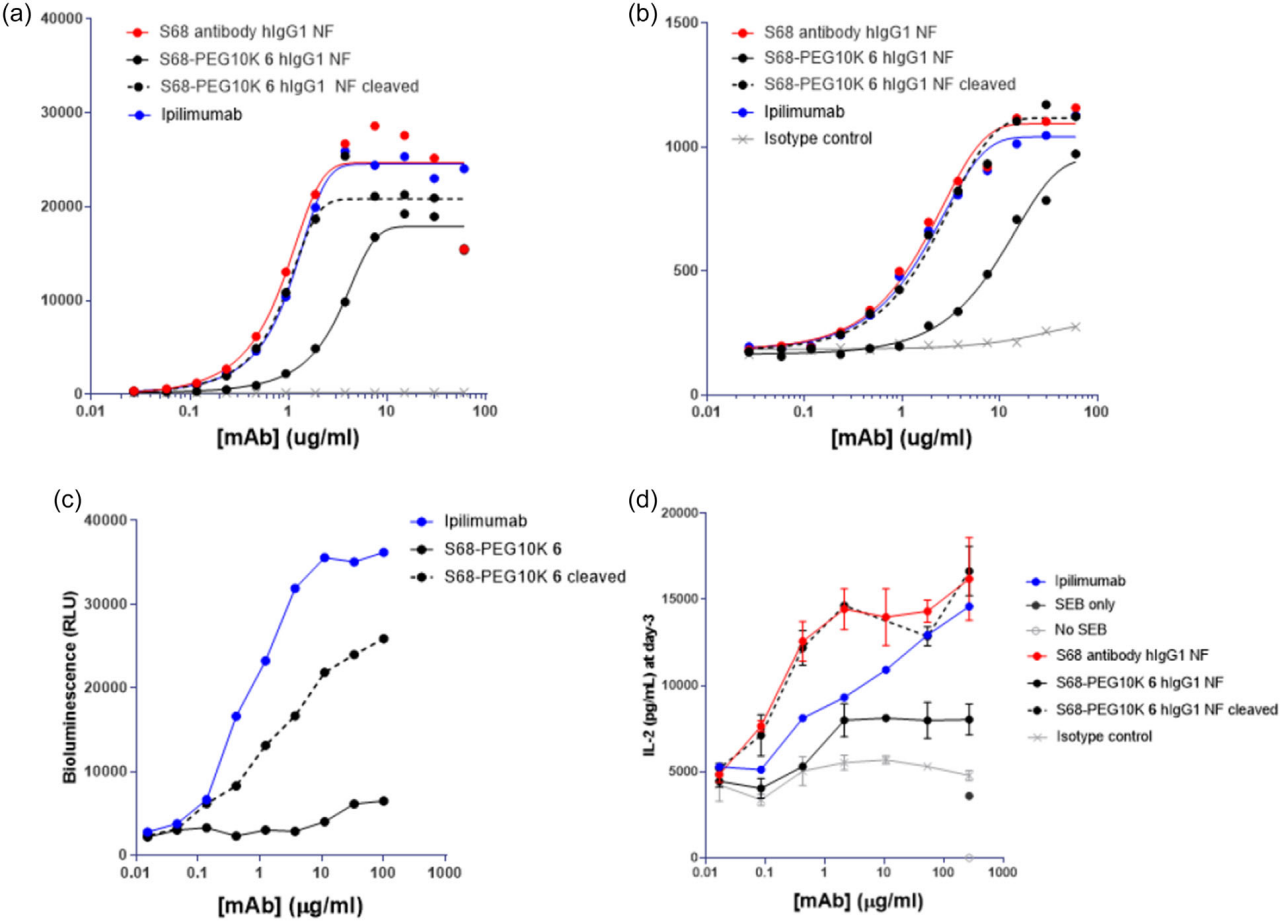
The activity of prodrugged and deprodrugged antibodies was characterized by an in vitro cellular binding and functional assays. The binding profile on a) CTLA4‐transfectant cell line and b) on activated CD4+ T cells is shown. c,d) (c) The CTLA4 blockade bioassay using cell propagation model and (d) IL‐2 production profile using SEB shown.

We decided to apply our masking technology to ipilimumab NF to assess if the technology can block the enhanced activity of ipilimumab. The genetic variant bearing the conjugation site at S68 and 10K PEG masking reagent (**6**) were used for the study. Similar to what is observed with prodrugged hIgG1 antibody, the incorporation of 10K PEG into the anti‐CTLA‐4 antibody hIgG1 NF resulted in a reduction of binding to CTLA‐4 expressing cells, and removal of PEG by matriptase restored binding (**Figure** **7**a,b). The prodrugged and deprodrugged antibodies in the hIgG1 NF backbone were further subjected to functional assays in vitro. The prodrugged antibody failed to demonstrate CTLA‐4 pathway blockade in a cell propagation model, while the activity of the unmasked antibody was detected in the system (Figure 7c). Likewise, the prodrugged antibody showed significantly reduced activity in SEC assays compared to the deprodrugged antibody (Figure 7d). The data support that PEG masking was effective in attenuating the NF version of ipilimumab. We then analyzed the binding kinetics of the prodrugged and deprodrugged antibodies to CTLA‐4 and Fcγ receptors (FcγRs). A mild level of binding reduction to CTLA‐4 was observed for the prodrugged version compared to the unmasked antibody (**Table** **6**, Figure S9, Supporting Information). The weak binding attenuation observed in SPR analysis, in contrast to the nearly complete functional attenuation, may be attributed to the nonideal behavior of PEGylated molecules, which can affect sensor surface interactions, as indicated by variability in R_max values. This variability likely arises from the flexible and dynamic nature of large, nondiscrete PEG chains, which can interfere with the accurate measurement of binding kinetics. These findings highlight the limitations of SPR in evaluating PEGylated antibodies. Although only minor binding attenuation was detected by SPR, the consistent reduction observed in more physiologically relevant systems—such as binding to primary cells and the abrogation of IL‐2 induction in PBMC assays—supports the conclusion that PEGylation of anti‐CTLA‐4 antibodies has a meaningful impact on biological activity. Although masking was intended to reduce the binding of antibodies to CTLA‐4, we also observed substantial binding attenuation of these antibody variants to FcγRs, including CD16 and CD64 (Figure S10, Supporting Information).

**Table 6:** Binding kinetics profiles of prodrugged and deprodrugged molecules.

Ligand	Analyte	Ka (1/Ms)	Kd (1/s)	KD [M]
S69 naked antibody	hCTLA4	3.10E + 05	2.70E‐02	8.60E‐08
S68‐PEG10K	hCTLA4	2.90E + 05	3.20E‐02	1.10E‐07
S68‐PEG10K cleaved	hCLTA4	2.90E + 05	2.40E‐02	8.40E‐08

## 3 In Vivo PK Characterization

In order to evaluate the exposure and track cleavage events in plasma and tissues, we measured the PK profile using MC38 tumor‐bearing C57BL/6 mice following a single dose administration of nonfucosylated human ipilimumab‐PEG in comparison with nonfucosylated ipilimumab. We administered the antibodies at a dose level of 10 mg kg^−1^ and collected serum and tissue samples at various time points. Two quantitative methods were employed to assess the exposure of the prodrugged and naked antibodies: ligand‐binding assay (LBA) and LC‐MS/MS. For LBA assays, the analyte was incubated with beads attached to an anti‐ipilimumab antibody. After immobilization, the captured ipilimumab‐containing species were detected using an anti‐human IgG Fc antibody to measure total monoclonal antibody (mAb). This method, using anti‐ipilimumab antibody as the capture reagent, enriches the totality of any ipilimumab‐containing species, including unconjugated antibody, mono‐ and bis‐PEG conjugated antibodies (total mAb). Total conjugates were measured using anti‐PEG as capturing reagents, and quantitation was performed using the anti‐human IgG1 Fc antibody. This method only captures PEGylated species, including mono‐ and bis‐PEGylated species; however, unconjugated antibody is eliminated due to the lack of a PEG moiety (total conjugate). For the LC‐MS method, we used the same capture reagents, while detection and quantitation were performed using multiple reaction monitoring.

The exposure data obtained with the two approaches showed excellent agreement, so we describe here the PK parameters obtained from the LBA method. While the clinical application of ipilimumab leverages intravenous (IV) dosing, we initially tested the intraperitoneal (IP) dosing route for the mouse study for technical ease and reduced damage to the laboratory animals. Serum samples were collected from drug‐administered animals and subjected to systemic exposure analysis. It was observed that ipilimumab NF conjugated with PEG10K is rapidly cleared from circulation following IP administration, while systemic levels of total mAb remain relatively stable (Figure S11, Table S3, Supporting Information). This discrepancy between total mAb and total conjugate levels suggests potential biotransformation within the peritoneal cavity prior to systemic absorption. However, due to the limited understanding of the absorption, distribution, metabolism, and excretion processes for biologics administered via the IP route, interpreting these findings remains challenging. Consequently, IP administration may not be the most suitable approach for accurately characterizing the systemic exposure of biologics, particulary chemically modified species.^[^ ^17^ ^]^

To rule out any potential deconjugation after IP administration and to allow for evaluation of PKs with clinical translational value, we decided to repeat the mouse PK study with the IV dosing route. For this study, plasma samples were collected at several time points and analyzed by immunocapture and LBA. In this study, ipilimumab NF‐PEG10K demonstrated greater exposure and duration of exposure, as evidenced by increased area under the curve (AUC) and mean residence time (MRT) in plasma, than nonPEGylated naked ipilimumab NF (**Table** **7**). This resulted in the plasma clearance of the prodrugged antibody being calculated to be several fold lower than the naked antibody. There was great agreement between the systemic exposure of total mAb and total conjugate, validating the stability of PEGylated molecules in circulation (Figure S12, Supporting Information).

**Table 7:** PK parameters of prodrugged molecule in plasma from C57Bl/6 mice bearing MC‐38 (IV).

Dosed articles	Species	Matrix	*T*_max_ [h]	*C*_max_ [nmol mL^−1^]	AUClast [nmol*h mL^−1^]	MRTlast [h]	T1/2 [h]	Vss (obs) [mL kg^−1^]	CL (obs) [mL/kg/Day]	Cavg (168 h) [nmol mL^−1^]
Ipilimumab hIgG1 NF	NA	Plasma	0.5	1074	33,261	45.2	34.3	97.6	46.7	198
S68‐PEG10	Total conjugate	Plasma	0.5	1238	110,878	68.9	110	54.9	8.24	660
S680PEG10K	Total mAb	Plasma	0.5	1156	108,806	191,916	141	61.9	7.36	648

The exposure profile of total mAb and total conjugate in tissues showed variable results. For total mAb, a delayed *T*_max_ of 4 h in tumors was observed for both prodrugged and naked antibodies, compared to a *T*_max_ of 0.25 h in the highly vascularized spleen and liver. The exposure duration of total mAb, as measured by MRT, was approximately the same in tumor (MRT 82 h), spleen (MRT 76 h), and liver (MRT 77 h) (**Table** **8**). We noted that the concentration of total conjugates was generally lower than the concentration of total antibody in these tissues, suggesting prodrug processing of administered antibodies after tissue uptake. The measured total conjugate level in the liver was the lowest, resulting in a greater discrepancy between total mAb and total conjugate in the liver compared to tumor and spleen, indicating high conversion to deprodrugged species in the liver.

**Table 8:** PK parameters of prodrugged molecule in tissues from C57Bl/6 mice bearing MC‐38 (IV).

Dosed article	Species	Matrix	*T*_max_ [h]	*C*_max_ [nmol mL^−1^]	AUClast [nmol*h mL^−1^]	MRTlast [h]	Cavg (168 h) [nmol mL^−1^]
Ipilimumab hIgG1 NF	NA	Tumor	4	88	9020	69.4	53.7
S68‐PEG10K	Total conjugate	Tumor	4	35.7	5252	81.2	31.3
S68‐PEG10K	Total mAb	Tumor	4	76	11,462	81.8	68.2
Ipilimumab hIgG1 NF	NA	Spleen	0.5	80.1	7448	71.3	44.3
S68‐PEG10K	Total conjugate	Spleen	0.5	44.4	5295	80.5	31.5
S68‐PEG10K	Total mAb	Spleen	0.5	70.3	7439	75.7	44.3
Ipilimumab hIgG1 NF	NA	Liver	0.5	91.5	3577	50.2	21.3
S68‐PEG10K	Total conjugate	Liver	168	7.71	1101	98.1	6.56
S68‐PEG10K	Total mAb	Liver	0.5	64	5345	77.1	31.8

Overall, IV administration of ipilimumab NF‐PEG10K resulted in the highest overall exposure (AUC) of total antibody and total conjugate in the tumor, followed by spleen and liver, suggesting efficient distribution of PEGylated species to the tissue, including the tumor. However, a reduced exposure of total conjugate compared to total mAb in these tissues was observed, indicating prodrug processing upon tissue uptake.

## 4 Conclusion

Therapeutic antibodies can be used to treat a variety of diseases, especially cancer and inflammatory conditions. Generally, a therapeutic antibody acts by binding with high specificity and affinity to its molecular target to initiate the cellular processes related to its therapeutic action. Masking is a potential strategy to mitigate any undesirable activity in nontargeted tissues, which could lead to an improved safety profile and PK profile by reducing TMDD.

We explored masking the therapeutic antibody ipilimumab by introducing a large PEG molecule to specific sites on the antibody. Unpaired cysteine mutations were introduced to various moieties of ipilimumab, particularly focusing on sites that are 1) solvent‐exposed and 2) within or adjacent to the CDRs or the Vernier zone. Through screening, we identified prodrugged variants that effectively block binding and immune‐activating functions while allowing for efficient cleavage and activity restoration upon exposure to a cleaving enzyme. To our surprise, the antibody showed substantially reduced binding to both the target and FcγRs. The prodrugged antibody demonstrated high stability in circulation and an improved systemic PK profile in a mouse model when administered via IV dosing. Although the exposure analysis of total mAb and total conjugate indicated prodrug processing and unmasking in the TME, the cleavage process was also indicated to take place in nontargeted tissues, including the liver and spleen. Although literature reports support the selective cleavage of the LSGR peptide by matriptase, which is overexpressed in the TME, we observed cleavage of the LSGR‐modified antibody in nontumor tissues, such as the liver and spleen.^[^ ^9^ ^b]^ This unexpected activity suggests that substrate specificity may be influenced by the structural context in which the peptide is incorporated into the antibody. Factors such as local conformation, steric accessibility, or interactions with off‐target proteases could contribute to nontumor‐specific cleavage. These findings highlight a potential limitation of the current masking strategy and underscore the need for further optimization to enhance tumor selectivity and minimize off‐target activation. As ipilimumab lacks murine cross‐reactivity, the observed extension in half‐life (T1/2) could be due to reduced Fc–FcγR interactions.

Although the reduced binding and functional attenuation of the prodrugged antibody, along with its stability in systemic circulation, might provide benefits in reducing undesirable activity in serum that could influence immune‐related adverse events, follow‐up studies are warranted to evaluate the consequences of unmasking events observed in the spleen and liver. Through our screening, we identified a conjugation site present in the framework region that enables efficient incorporation of the masking reagent without causing aggregation. This technology could provide a universal chemical approach to attenuate the binding and functions of therapeutic antibodies.

## 5 Experimental Section

5.1

5.1.1

##### Matriptase Cleavage of Linker Moiety

Prodrugged antibody was incubated with human matriptase (30:1 molar ratio, R&D system, 3946‐SE‐010) in 100 mM Tris buffer, pH7.6 at 37C. At each time point, 10 μL of sample was mixed with 10 μL quenching buffer (100 mM phosphate buffer with 4 M GdnCl and 0.4M TCEP, pH2.5) to simultaneously deactivate the enzyme and reduce the prodrugged antibody. The quenched sample was analyzed by LC/MS. MS data was used to calculate DAR of conjugated antibodies.

##### Binding to Activated CD4+ T‐Cells

Serial dilutions of prodrugged and deprodrugged antibody were tested, and binding was detected using a fluorescently labeled anti‐human IgG1 secondary antibody. Flow cytometric analysis was performed on a BD Canto flow cytometer, collecting at least 10,000 events per sample. The geometric mean fluorescence intensity was determined using FlowJo analysis software. The binding profiles of both test articles were compared to that of unprodrugged ipilimumab.

##### IL‐2 Secretion Assay

The activity of prodrugged and deprodrugged antibodies was characterized by an in vitro functional assay using SEB. Fresh PBMCs were isolated from two healthy human donors and treated with several concentrations of prodrugged and deprodrugged antibodies. Simultaneously, a suboptimal concentration of SEB was added to stimulate the cells. T‐cell activation was monitored by measuring secretion of the cytokine IL‐2 after day 3 of incubation.

##### Mice

All animal procedures were performed in accordance with animal protocols that were approved by the Institutional Animal Care and Use Committee. The animal care and use program at BMS is fully accredited by The Association for Assessment and Accreditation of Laboratory Animal Care International. The in vivo experiments were performed in the following mouse strains: Six‐to‐eight‐week‐old C57BL/6 female mice (the Jackson Laboratory). Mice were maintained under pathogen free conditions and randomly assigned to each experimental group.

##### Cell Lines

MC38 (RRID: CVCL_B288) cell line from ATCC was cultured at 37 °C in DMEM (Gibco), respectively supplemented with 10% fetal bovine serum (Invitrogen). The cells were verified to be mycoplasma‐free and cultured with in the medium supplemented with 10% FBS at 37C and 5% CO_2_ atmosphere.

##### PK Study Sampling with MC38 Bearing Mouse Model

MC38 cells were resuspended in PBS and subcutaneously injected into the flank of C57/BALB/c mice. Treatment was initiated when tumors reached a volume of 100 mm^3^. The prodrugged antibody and isotype control were diluted with PBS and administered at a dose of 10 mg kg^−1^. Antibodies were dosed via IV or IP injection single dose. Serum or plasma samples were collected at 0.5, 4, 24, 96 and 168 h time points by serial or terminal bleeds. Tissue samples were collected at 0.5, 4, and 168 h time points. Three mice per each time point was used for PK analysis.

##### Total Antibody and Total ADC Determination by Enzyme‐Linked Immunosorbent Assay

Streptavidin 96‐well multiarray plate (Meso Scale Discovery) was first coated with the capture reagent by incubating with Biotinylated anti‐Ipilimumab (in house) and shaking at 450 rpm at room temperature for 1 h. After the incubation, the plates were washed three times. An 8 point standard curve was prepared by spiking naked or prodrugged antibodies into the matrix (1:9 mouse blood:Rexxip A, serum or naïve tissue lysate) from a range of 0.15–2500 ng ml^−1^ along with QCs at 2, 20, 200, and 2000 ng ml^−1^. The tissue samples were first homogenized in naïve mouse serum containing protease inhibitor cocktail using a bead mill homogenizer (Omni International) to generate the lysates. Finally, the standard curve, QC, and the samples were diluted tenfold in assay diluent (blocker blotto). 25 μl of these samples were added to MSD plate coated with capture reagent and incubated for 1.5 h. After the incubation, the plates were washed, and detection reagent was added and incubated for 1 h with gentle shaking. The detection reagent for total antibody assay was anti‐human H + L sulfotag for plasma and 10C7 sulfotag for tumor, spleen, and liver homegenate. The detection reagent for total antibody‐drug‐conjugate (ADC) assay was a sulfo tag labeled anti PEG antibody (AGP4 IgM). After the final incubation, the plates were washed and then 150 μl of Read Buffer A (Meso Scale Discovery) was added and the plates were immediately read on MSD Quickplex 120 instrument. The ECL signals measured were analyzed using the MSD Discovery Workbench software to determine the concentration of analyte in the samples. The blood:Rexxip samples were multiplied by hematocrit factor (17.4) to determine the concentration in serum, while the tissue samples were normalized to the weight of tissue.

## Conflict of Interest

The authors declare no conflict of interest.

## Supporting information

Supplementary Material

## Data Availability

The data that support the findings of this study are available in the supplementary material of this article.
