# Supplementary material for: Design and Characterization of Prodrugged Anti‐CTLA‐4 Antibodies
Source: Chembiochem. 2025 Oct 28;26(22):e202500304. doi: 10.1002/cbic.202500304 (PMC12631005; doi:10.1002/cbic.202500304)
Supplement: Supplementary file 1 — Supplementary Material [file CBIC-26-e202500304-s001.pdf]

SUPPORTING INFORMATION

Design and Characterization of Prodrugged Anti-CTLA-4 Antibodies

Sayumi Yamazoe*, Mary Huber, Srikanth Kotapati, Rahima Akter, Aarti Jashnani, Suprit Deol, Christine Bee, John Engelhart, Yam B Poudel, Stanley Krystek, John Haugner, Mohan Srinivasan, Arvind Rajpal, Yong Zhang, Pavel Strop, and Chetana Rao

WILEY-VCH

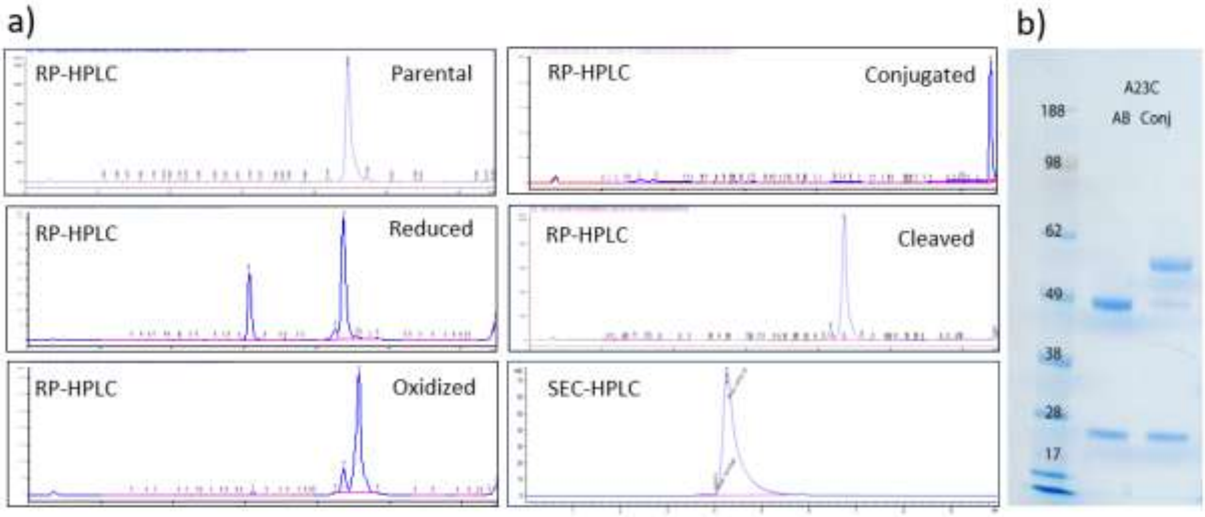

Figure S1: Monitoring reactions and characterization of A23C construct-conjugated to PEG reagent. Reverse-phase (RP) chromatograph shows separation of heavy and light chains upon partial reduction, chain re-folding by oxidation, conjugation with hydrophilic PEG reagent. Digestion of the conjugated molecule by matriptase resulted in the retention time shift indicative of reverting to unmasked antibody. Size-exclusion column chromatography showed high monomer purity. SDS-PAGE shows the shift in band size after conjugation, confirming attachment of bulky PEG reagent.

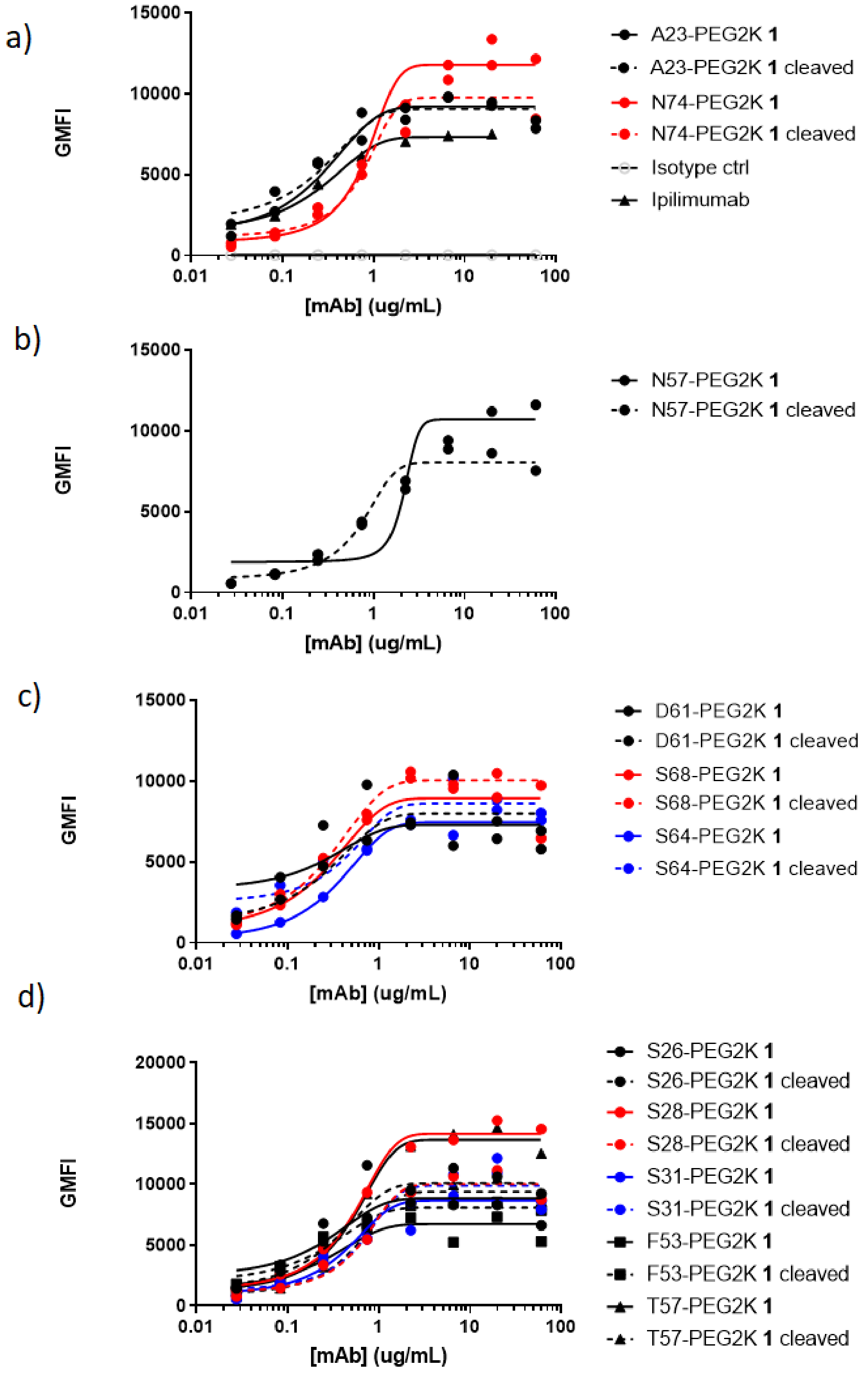

Figure S2: Binding of prodrugged and unmasked formats of anti-CLTA antibodies on cell lines engineered to overexpress CTLA4. Binding profiles were shown for the constructs bearing cysteine mutation at heavy chain framework (a), heavy chain CDRs (b), light chain CDRs (c), and light chain framework (d).

						Matriptase Digestion Rate (RFU/min)					
						Human			Murine		
	P4	P3	P2	P1		pH 7.3	pH 6.5	pH 6.0	pH 7.3	pH 6.5	pH 6.0
Ac	L	S	G	R	AMC	454	150	42	538	189	57
Ac	L	S	G	K	AMC	221	70	17	184	56	17

Figure S3. Matriptase cleavage efficiency of LSGR and LSGK peptides was measured. The cleavage kinetics were assessed for both human and murine matriptase. Both peptides showed comparable profiles with human and murine matriptase. Although cleavage in acidic pH was somewhat compromised, the LSGR peptide exhibited higher cleavage kinetics.

WILEY-VCH

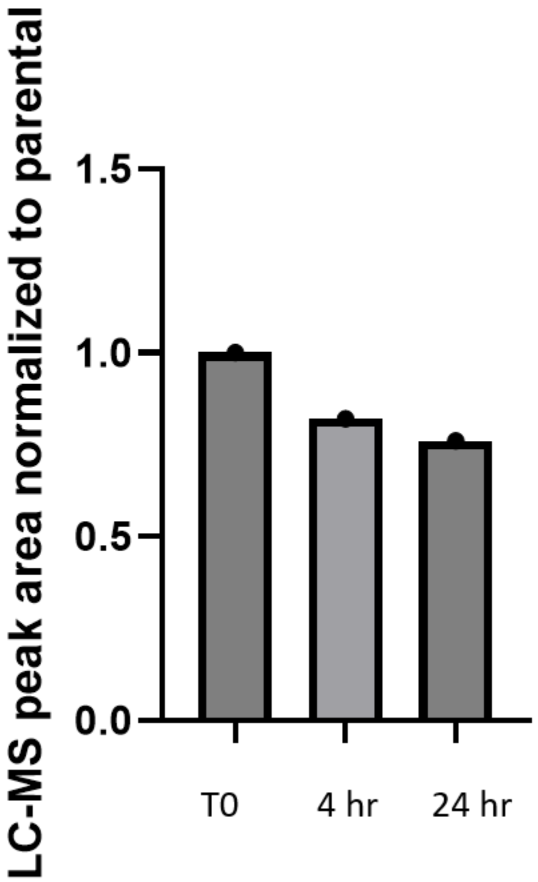

Figure S4. LC-MS of LSGR peptide incubated in 50% human serum at pH7.3.

WILEY - VCH

Table S1. Conjugated products using PEG5K and PEG10K reagents and characterization data

Site (Kabat)	Chain	Position	Linker	Aggregation	DAR	Product
A23	Heavy	FR	PEG5K (**2**)	<1%	1.93	A23-5K **2**
A23	Heavy	FR	PEG10K (**3**)	<1%	1.99	A23-10K **3**
S68	Light	FR	PEG5K (**2**)	4%	1.96	S68-5K **2**
S68	Light	FR	PEG10K (**3**)	<1%	1.99	S68-10K **3**
A23	Heavy	FR	PEG5K (**2**)	<1%	1.96	A23-5K **2** cleaved
A23	Heavy	FR	PEG10K (**3**)	<1%	1.98	A23-10K **3** cleaved
S68	Light	FR	PEG5K (**2**)	<1%	1.96	S68-5K **2** cleaved
S68	Light	FR	PEG10K (**3**)	3.6%	1.96	S68-10K **3** cleaved

WILEY-VCH

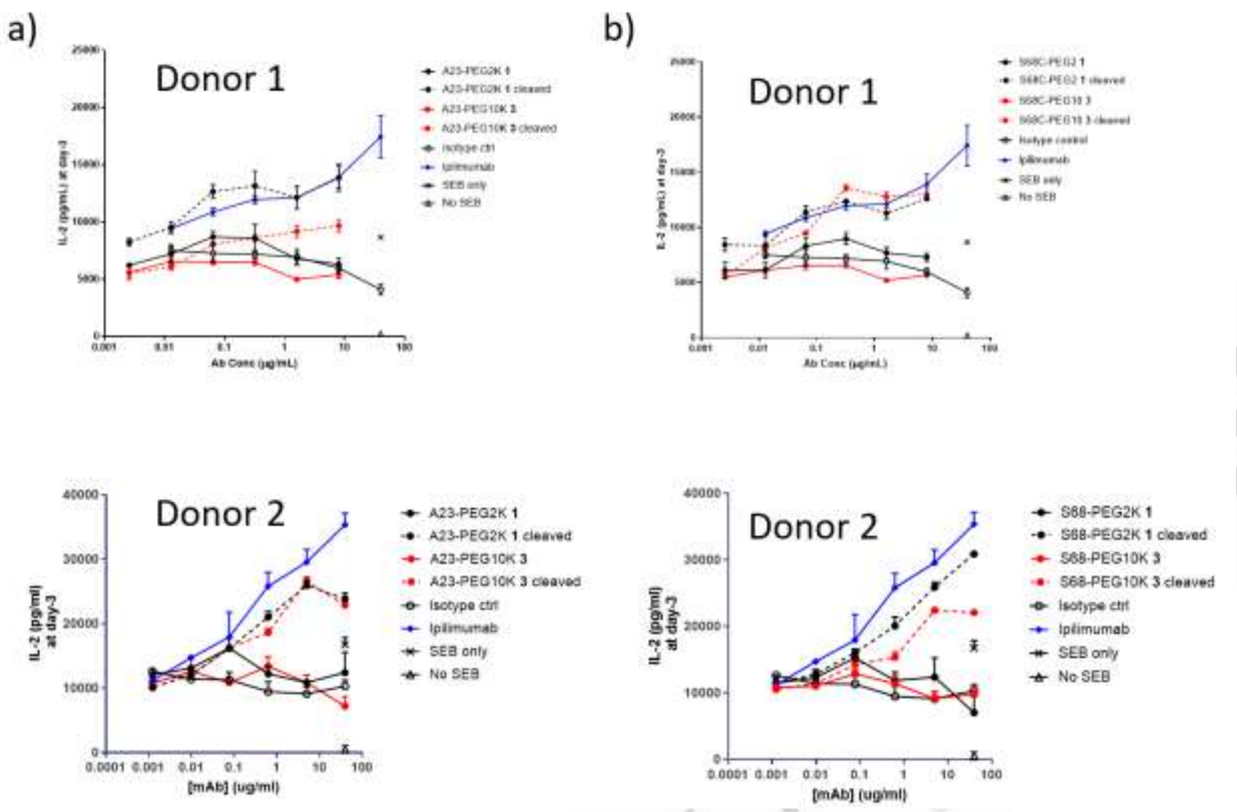

Figure S5. The activity of prodrugged and de-prodrugged antibodies was characterized by an in vitro functional assay using Staphylococcal enterotoxin B (SEB). a, b) IL-2 production profile of prodrugged and un-prodrugged molecules bearing masking group at A23 (b) and S68 (c) is shown.

WILEY

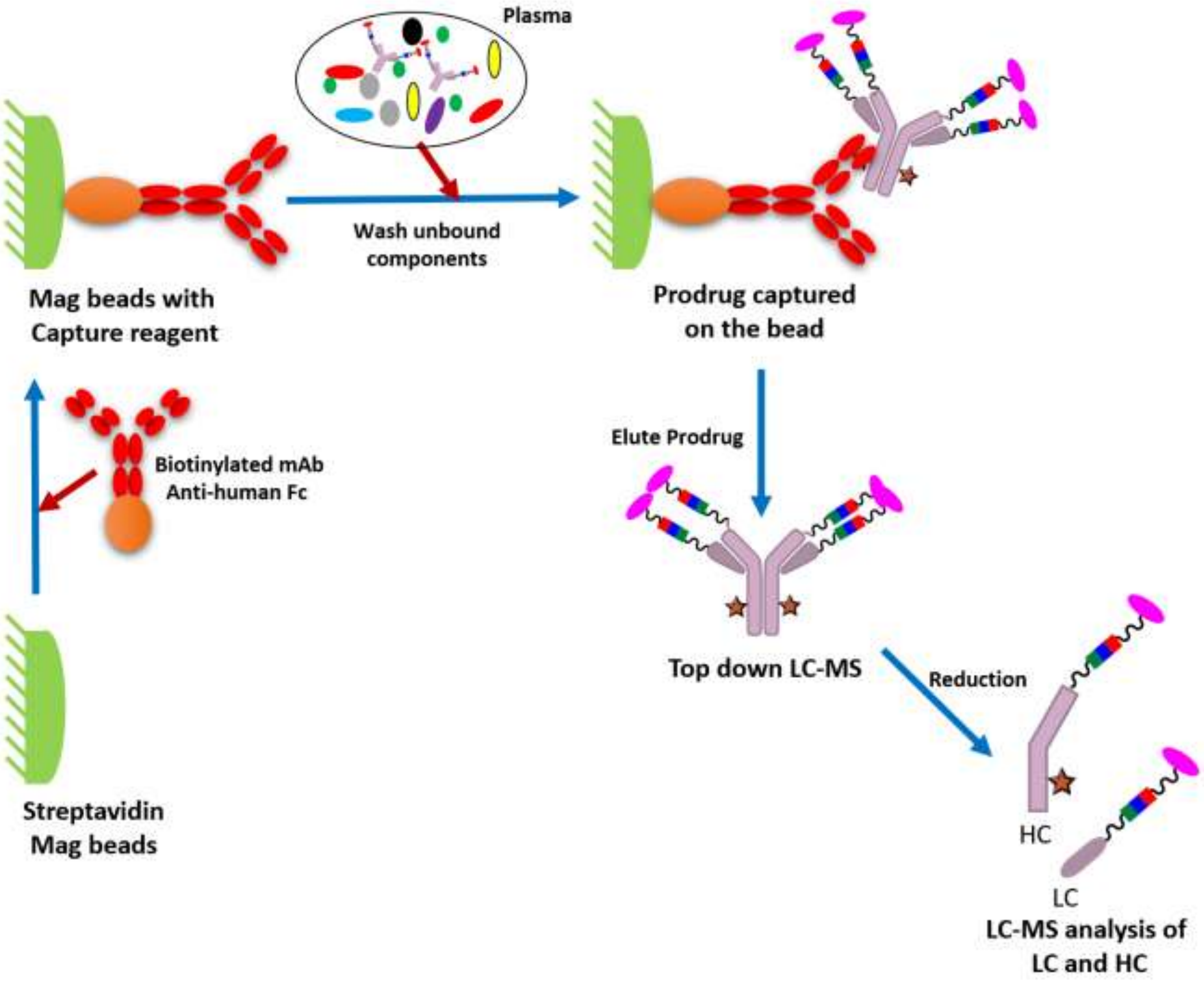

Figure S6. Bioanalytical characterization method used to evaluate serum stability of prodrugged molecules. Both PEGylated and unmasked formats were tested.

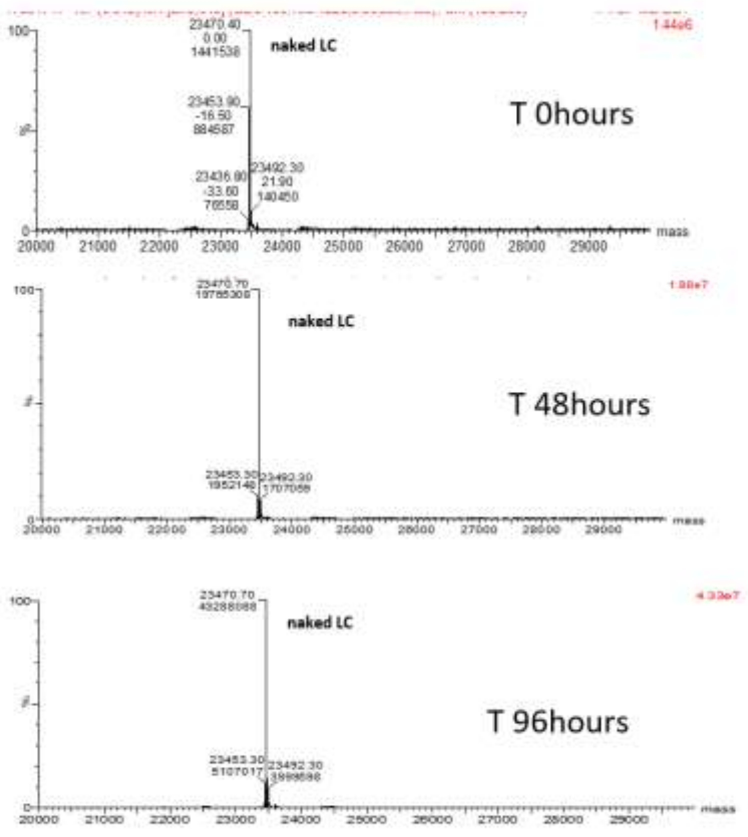

Figure S7. In-serum biotransformation analysis of the prodrugged molecule with PEG5K at the S68 position, deconvoluted mass spectra of samples from 0, 2, and 4 days are shown. Due to poor ionization of the 5K PEG, only the unconjugated light chain was observed.

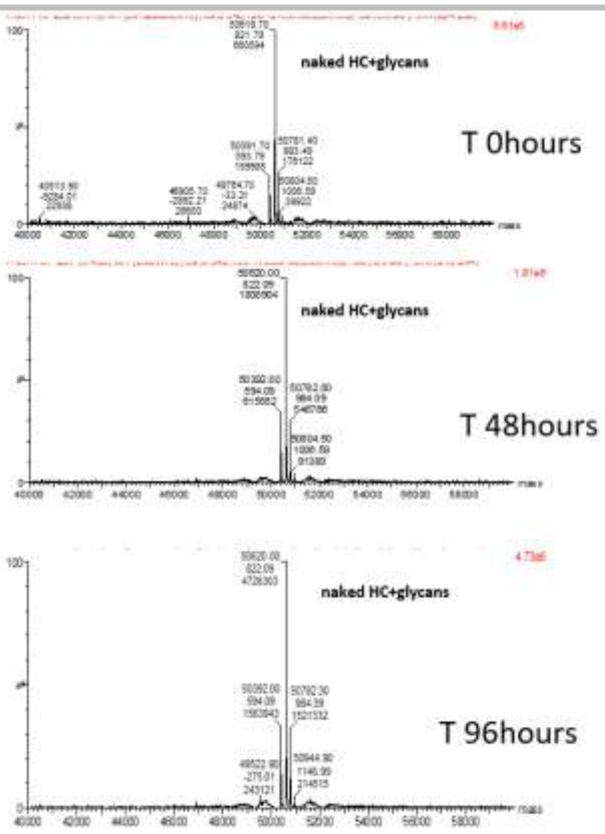

Figure S8. In-serum biotransformation analysis of the prodrugged molecule with PEG5K at the A23 position, deconvoluted mass spectra of samples from 0, 2, and 4 days are shown. Due to poor ionization of the 5K PEG, only the unconjugated light chain was observed.

Table S2. List of prodrugged and de-prodrugged molecules and characterization data

Site (Kabat)	Format	Linker	Aggregation	DAR	Product
A23	Prodrugged	PEG2K (**4**)	<1%	1.93	A23-2K **4**
A23	Prodrugged	PGE5K (**5**)	<1%	2.0	A23-5K **5**
A23	Prodrugged	PGE10K (**6**)	<1%	1.97	A23-10K **6**
A23	De-prodrugged	PEG2K (**4**)	2.1%	2.0	A23-2K **4** cleaved
A23	De-prodrugged	PGE5K (**5**)	1.4%	2.0	A23-5K **5** cleaved
A23	De-prodrugged	PGE10K (**6**)	1.2%	2.0	A23-10K **6** cleaved
S68	Prodrugged	PEG2K (**4**)	<1%	2.0	S68-2K **4**
S68	Prodrugged	PGE5K (**5**)	<1%	2.0	S68-5K **5**
S68	Prodrugged	PGE10K (**6**)	<1%	2.0	S68-10K **6**
S68	De-prodrugged	PEG2K (**4**)	<1%	1.95	S68-2K **4** cleaved
S68	De-prodrugged	PGE5K (**5**)	<1%	2.0	S68-5K **5** cleaved
S68	De-prodrugged	PGE10K (**6**)	<1%	2.0	S68-10K **6** cleaved

WILEY

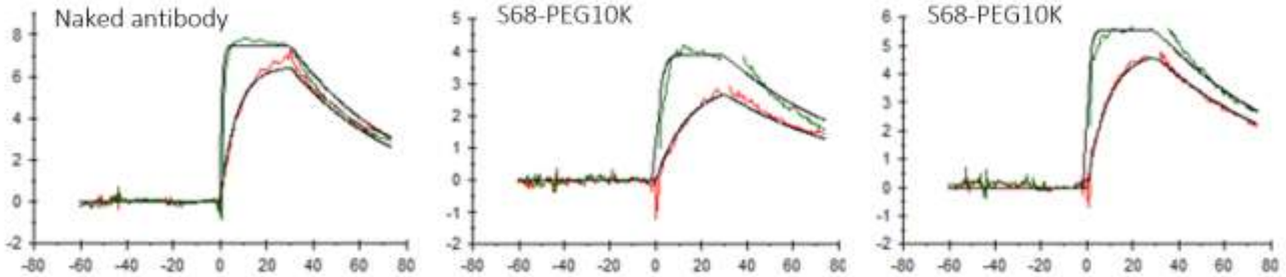

Figure S9. Binding profiles of prodrugged and de-prodrugged molecules to CTLA4 were analyzed. The sensorgram of SPR with different CTLA4 capture levels is shown (low/red and high/green)

WILEY-VCH

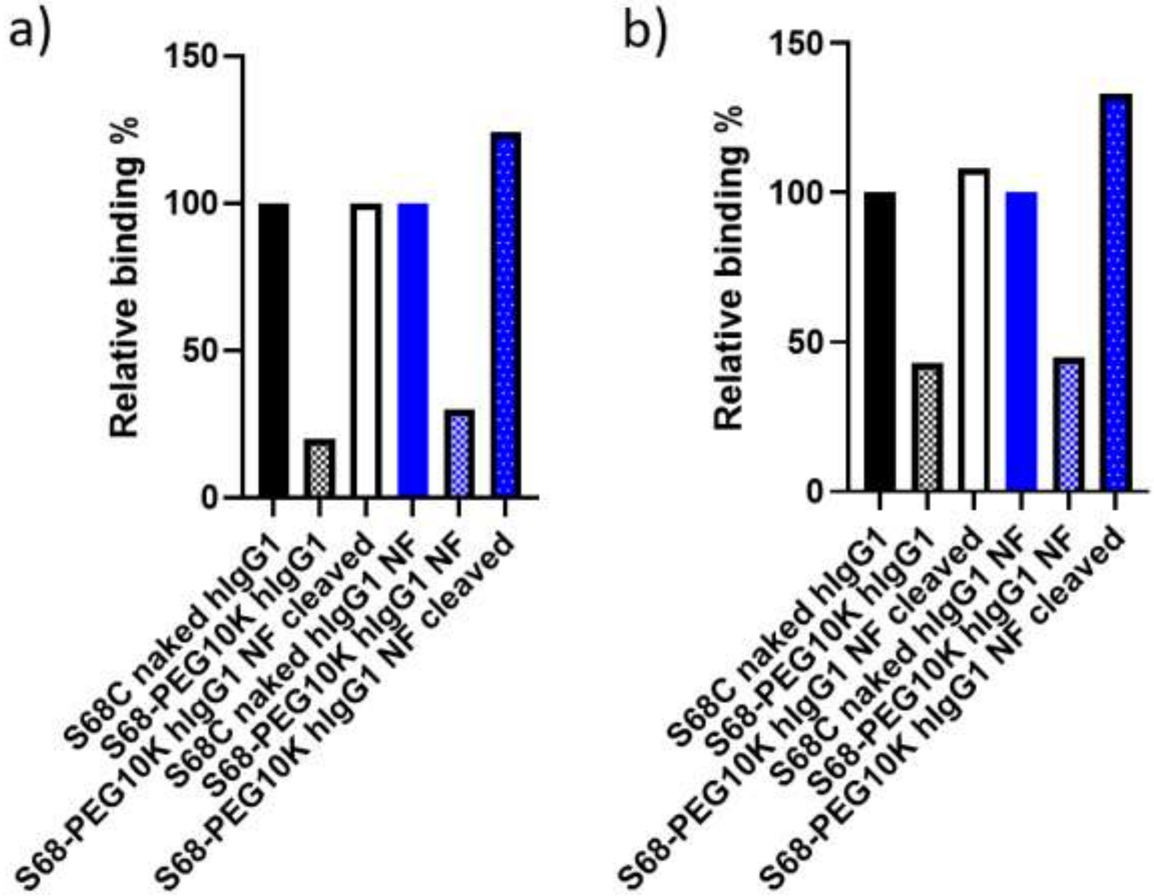

Figure S10. Partial binding attenuation to FcγRs of the prodrugged molecules was observed. The relative binding, averaged over all concentrations, is shown for the parental antibody, PEGylated, and unmasked formats. The binding profile was measured against CD16 (a) and CD64 (b).

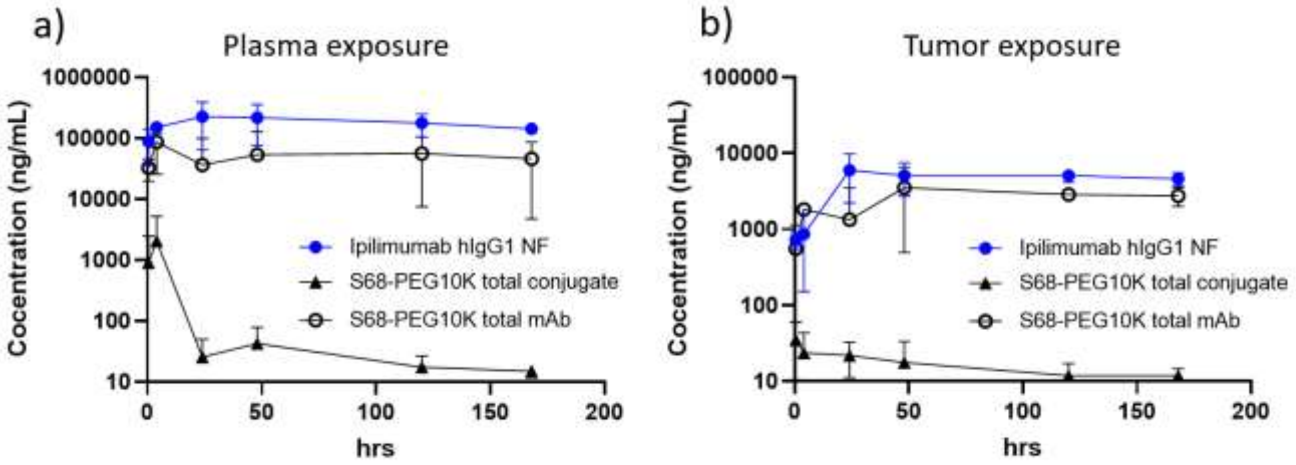

Figure S11. Exposure profiles of Ipilimumab hIgG1 NF and prodrugged antibodies. a-c PK profiles in plasma (a), and tumor (b) after a single IP administration of mAb and conjugates in mice.

WILEY-VCH

Table S3. PK parameters of prodrugged molecule in tumor from C57Bl/6 mice bearing MC-38 (IP)

Dosed article	Species	Matrix	Tmax	Cmax	AUClast	MRTlast	T1/2	Vss (obs)	CL (obs)	Cavg (168h)
			h	nmol/mL	nmol*h/mL	h	h	mL/kg	mL/kg/Day	nmol/mL
Ipilimumab higG1 NF	N.A.	Plasma	24	1434	204493	45.2	394	47.3	1.99	1215
S68-PEG10K	Toal conjugate	Plasma	4	23.6	176.7	18.8	74.5	37724	8420	1.1
S68-PEG10K	Total mAb	Plasma	4	860	82532	79.4	161			491

Dosed article	Species	Matrix	Tmax	Cmax	AUClast	MRTlast	Cavg (168h)
			h	nmol/mL	nmol*h/mL	h	nmol/mL
Ipilimumab higG1 NF	N.A.	Tumor	120	45.5	6234	92.0	37.1
S68-PEG10K	Toal conjugate	Tumor	0.5	0.27	20.6	69.2	0.124
S68-PEG10K	Total mAb	Tumor	48	23.6	2687	85.3	15.9

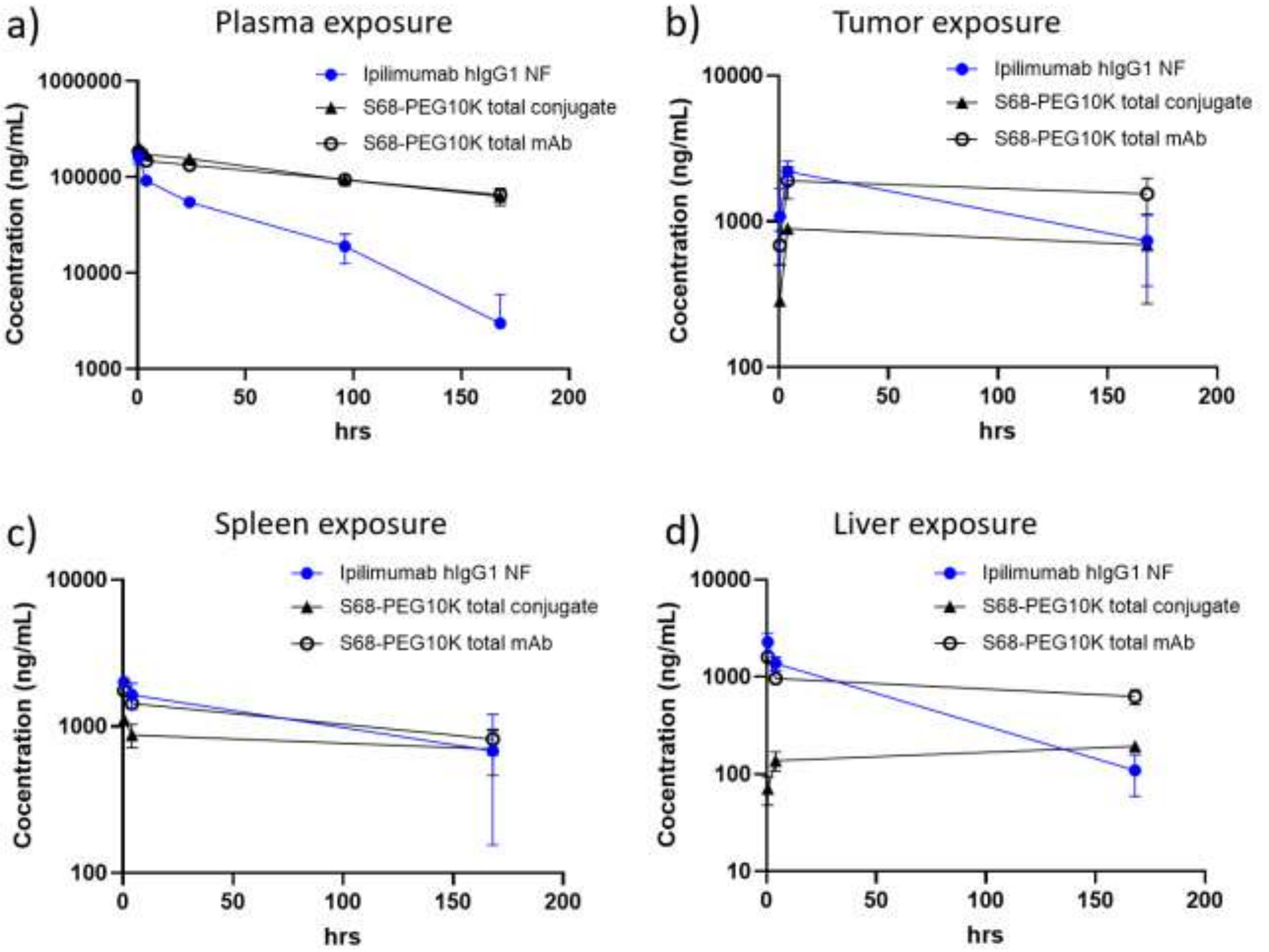

Figure S12. Exposure profiles of Ipilimumab hIgG1 NF and prodrugged antibodies. a-c PK profiles in plasma (a), tumor (b), spleen (c) and liver (d) after a single iv administration of mAb and conjugates in mice.

ADC conjugation. Antibodies having selected Cys substitutions were transiently expressed in Expi-CHO cells and purified using standard protocls with protein A chromatography. Purified antibodies were treated with an excess (10-30 molar equivalents) of a reducing agent TCEP at 37C for 1-3 hours in a buffered aqueous solution containing 2 mM EDTA. The TCEP was removed by passing the reduced variant antibody through a PD-10 column or Sephadex G-25 depending on the scale of bioconjugation. The reduction of the antibody was confirmed on an analytical reverse phase HPLC system. The purified, reduced antibody was treated with an excess of dhAA at room temperature for 1-3 hours. The re-oxidation of the antibody was monitored on an analytical reverse phase HPLC and aggregation levels on analytical size-exclusion column. After reduction and re-oxidation as described above, the antibody in buffered aqueous solution was treated with 3 molar equivalents of a linker per thiol of antibody containing maleimide. The reaction was allowed to proceed for 2 hours at room temperature or 4C overnight. Afterwards, the conjugate was purified by protein A, ion exchange, or size exclusion column chromatography. Analytical test was performed by RP-HPLC and SEC to assess conjugation and aggregation levels.

SPR binding. All SPR data was collected on a Biacore T200 instrument (Cytiva). Human CTLA4 fused to an AviTag was captured on a C1 chip with anti-Avi pAb. For kinetics measurements, the Yervoy (Ipilimumab) samples were applied over the captured antigen at concentrations ranging from 0.9 nM to 2 μM in HBS-P running buffer (0.01 M HEPES, pH 7.4, 0.15 M NaCl, 0.005% v/v Surfactant P20) at 37°C. The chip surface was regenerated between cycles with two 30-second pulses of 3M MgCl2. To dilute out avidity, CTLA4 was captured at two different densities.
For FcγRs binding, diluted antibody samples were injected over a Protein A sensor chip surface (Cytiva) for 30 seconds at 30 μL/min to an average capture level of about 250 RU. FcγRs were then injected at 30 μL/min at concentrations ranging from 1.4 to 10,000 nM for 120 seconds, followed by 120 seconds of dissociation in running buffer. Kinetic traces of reference-subtracted sensorgrams were fit to a 1:1 binding model using Biaevaluation software.

Matriptase digestion rate measurement. A matriptase cleavage assay was performed using a substrate linked to 7-Amino-4-methylcoumarin (AMC) to measure matriptase activity. Matriptase was incubated with the AMC-linked substrate (Ac-LSGX-AMC) in an appropriate buffer. The release of AMC, which emits fluorescence, was monitored using a fluorometer at excitation and emission wavelengths of 360 nm and 480 nm, respectively. The increase in fluorescence over time (RFU/min) was recorded, and the specific activity of matriptase was calculated as pmol/min/μg using a conversion factor derived from an AMC calibration standard.

Peptide Stability Assay. The stability of LSGR and LSGK peptides was assessed by incubating the peptides in 50% human serum at pH 7.3. The incubation was carried out at 37°C to mimic physiological conditions. At specified time points (0 hours, 4 hours, and 24 hours), aliquots were taken from the incubation mixture. The quantification of the peptides was performed using a Waters ZQ 2000 single quadrupole mass spectrometer. Chromatographic separations were achieved using analytical HPLC with a Waters Acquity SDS system, employing the following method: linear gradient of 2% to 98% solvent B over 1.7 minutes; UV visualization at 220 nm; column: BEH C18, 2.1 mm x 50 mm, 1.7 μm particle (heated to 50°C); flow rate: 0.8 mL/min; mobile phase A: 100% water, 0.05% TFA; mobile phase B: 100% acetonitrile, 0.05% TFA. Positive ESI MS data were acquired from m/z 135 to m/z 1000. The peptides were detected and quantified based on their UV absorption profiles. To determine the stability of the peptides, the levels of LSGR and LSGK at 4 hours and 24 hours were normalized to their respective quantities at the initial time point (T0).

WILEY-VCH
